# Approval of AI-Based Medical Devices in China From 2020 to 2025: Retrospective Analysis

**DOI:** 10.2196/85538

**Published:** 2026-03-18

**Authors:** Lingli Zhang, Jianzhou Yan

**Affiliations:** 1 School of International Pharmaceutical Business, China Pharmaceutical University, 639 Longmian Avenue, Jiangning District, Nanjing, 211198, China, 86 02586185038; 2 Research Center of National Drug Policy and Ecosystem, China Pharmaceutical University, Nanjing, China

**Keywords:** medical device, artificial intelligence, AI, artificial intelligence–based medical device, AI-based medical device, medical device approval, regulatory science, China, deep learning, radiology

## Abstract

**Background:**

Artificial intelligence–based medical devices (AIMDs) have emerged as transformative technologies in modern health care. However, comprehensive analysis of recent approval trends and characteristics of AIMDs in China remains limited.

**Objective:**

This study aimed to provide an up-to-date overview of AIMDs approved in China up to June 2025.

**Methods:**

We conducted a search of the Drugdataexpy database to identify AIMDs approved up to June 30, 2025, using artificial intelligence–related keywords in the “structural composition” and “intended use” fields. After manual verification and exclusion of non-AIMDs, we collected key characteristics, including name, manufacturer, approval date, risk class, clinical evaluation pathway, medical specialty, data source, review pathway, and algorithm type. Statistical analysis encompassed descriptive statistics and trend analysis. We used the Fisher exact test and Pearson chi-square test to assess the associations between risk class and categorical variables.

**Results:**

A total of 154 AIMDs were identified since the first approval in 2020, with annual approvals increasing from 9 in 2020 to 45 in 2024 (a 49.53% compound annual growth rate), although the 20 approvals in the first half of 2025 suggest a potential moderation in pace. Most AIMDs (123/154, 79.9%) were categorized as class III, and the risk class was significantly associated with approval year (*P*=.03), manufacturer location (*P*=.03), and medical specialty (*P*=.004). Of the 123 class III devices, 19 (15.4%) were approved through innovation review, and 2 (1.6%) each were approved through priority and emergency approval. Deep learning was the dominant algorithm (143/154, 92.9%). Radiology dominated the field (106/154, 68.8%), with computed tomography serving as the primary data source (96/154, 62.3%), particularly for applications in pulmonary nodule detection and cardiovascular assessment. Clinical trials were the primary evaluation pathway for 76.6% (118/154) of all AIMDs. This approach was predominant for class III devices (116/123, 94.3%), whereas most class II devices (21/31, 67.7%) used a clinical exemption pathway. Market concentration was evident, with the top 4 manufacturers accounting for 38.3% (59/154) of all approvals and geographically clustered in major innovation hubs such as Beijing, Shanghai, Shenzhen, and Hangzhou.

**Conclusions:**

China’s AIMD ecosystem is experiencing growth, heavily focused on radiology and computed tomography–based solutions within a risk-proportionate regulatory framework. The market is characterized by significant manufacturer and geographic concentration.

## Introduction

Artificial intelligence (AI) has emerged as a transformative force in modern health care, offering unprecedented capabilities in data analysis, pattern recognition, and clinical decision support [1,2]. Among its many applications, AI-based medical devices (AIMDs) have gained substantial traction, particularly in medical imaging, diagnostics, and predictive analytics [3]. These technologies promise to enhance diagnostic accuracy; reduce physician workload; and, ultimately, improve patient outcomes [4].

Worldwide, regulatory authorities have responded to the rapid development of AIMDs by establishing frameworks to ensure their safety, efficacy, and clinical utility [5-8]. In the United States, the Food and Drug Administration approved 691 AI- and machine learning (ML)–based medical devices by October 2023, with most cleared through the 510(k) pathway and concentrated in radiology and cardiology [9,10]. Similarly, Europe has witnessed a substantial increase in CE-marked AIMDs, with approval numbers growing significantly since 2015 [11].

China, as a major player in both AI innovation and health care digitalization, has also made significant strides in this domain [12,13]. The National Medical Products Administration (NMPA) approved its first AIMD in 2020. Since then, the number of approvals has grown steadily, reaching 59 by mid-2023, all categorized as class III [14]. In China, medical devices are categorized into 3 classes based on increasing risk: class I (low risk), class II (medium risk), and class III (high risk). This classification dictates the regulatory pathway for market approval. Class III devices, deemed to pose the highest risk, require centralized registration approval from the NMPA itself and are subject to the most stringent review of clinical evidence. Class II devices may receive approval from provincial-level medical product administrations. AIMDs in China are typically categorized as class II or class III. The classification is determined by the algorithm’s maturity and its role in clinical decision-making. Specifically, AIMDs with lower algorithm maturity are managed as class III devices if they are intended for auxiliary decision-making. Conversely, if they are intended for non–decision support tasks, they are managed as class II devices. In China, the clinical evaluation pathway for medical devices consists of 3 types: clinical trials, clinical comparison with similar products, and exemption from clinical evaluation. The clinical exemption pathway relies on the *Catalogue of Medical Devices Exempt From Clinical Evaluation* issued by the NMPA [15].

In recent years, the NMPA has actively refined and optimized its review processes for AIMDs. Key efforts include issuing evaluation guidelines, clarifying regulatory classifications, developing clinical evaluation guidance for specific product types, and introducing comprehensive support measures spanning the entire product life cycle [16-19]. These advancements have spurred a new wave of submissions and approvals, fostering a more structured and efficient ecosystem for AIMD market entry. Despite this progress, the literature to date primarily documents AIMD approvals in China up to 2023. There remains a lack of comprehensive analyses covering the most recent developments, particularly those in 2024 and 2025—a period likely marked by regulatory maturation and increased approvals. This study adopted innovation diffusion theory [20] as its primary analytical lens. This theory posits that the adoption rate and pattern of an innovation are influenced by its perceived attributes, including relative advantage, compatibility, complexity, trialability, and observability, within a specific social system.

Therefore, this study aimed to provide an up-to-date analysis of AIMDs approved in China as of June 2025. By examining trends in approval numbers, device characteristics, and regulatory pathways, it may offer insights into the evolving AIMD landscape in China and its implications for future health care innovation.

## Methods

### Data Source and Search Strategy

To identify AIMDs approved up to June 30, 2025, we searched the Drugdataexpy database [21]. As this is a Chinese-language database, the keyword search was performed using the Chinese equivalents of the following English-language terms in the database fields for “structural composition” and “indications” or “intended use”: “deep learning,” “machine learning,” “neural network,” “artificial intelligence,” or “algorithm.” The NMPA’s official website, while providing free access to regulatory announcements, lacks keyword-based filtering capabilities in its search function, thereby limiting efficient retrieval of AIMDs.

This study included AIMDs regulated under the Guidelines for Registration Review of Artificial Intelligence Medical Devices [16], encompassing stand-alone software as a medical device and software in a medical device (SiMD) where AI is a core component. After manual review of full-text descriptions, we excluded records that did not meet the AIMD definition. We also removed entries corresponding solely to routine administrative renewals without changes to the device’s core AI software or intended use. Substantive approvals for major changes or modifications to existing devices (eg, the addition of new AI functionality) were retained. Details of the search strategy and process are provided in Multimedia Appendix 1. To minimize omissions, we reran the search for each retained product name to capture devices manufactured by other companies. Our results were cross-validated against the AIMDs approved before July 2023 as listed in the study by Liu et al [14]. Ambiguous entries were checked against the corresponding NMPA evaluation reports or the manufacturers’ websites.

### Data Extraction

For each eligible AIMD, we extracted the approval number, device name, manufacturer, approval date, structural composition, intended use, risk class, clinical evaluation pathway (clinical trials, clinical comparison with similar products, or exemption from clinical evaluation), and review pathway (innovative, priority, or emergency special review vs standard). From these fields, we derived the city of the manufacturer, medical specialty, body area, and data source of the AIMD. The medical specialty for each AIMD was assigned based on the device’s registered intended use and its core function. For devices with applications relevant to multiple specialties, the specialty was determined by the primary clinical context and decision support role stated in the indications for use. The body area for each device was functionally classified into nonoverlapping categories based on the primary anatomical structure and type of clinical analysis specified in the device’s intended use: “heart” referred to devices targeting cardiac muscle, chambers, or electrophysiology (eg, electrocardiogram analysis); “cardiovascular” referred to devices focused on the vascular system (eg, computed tomography [CT] or magnetic resonance angiography); “lung” referred to devices analyzing lung parenchyma (eg, nodule detection); and “thorax” referred to devices assessing nonparenchymal structures within the thoracic cavity (eg, rib fracture detection). Algorithm type was determined through a tiered review: it was first identified from the “structural composition” and “intended use” fields. If not specified there, the full registration dossier was consulted for explicit technical descriptions.

To facilitate downstream data processing, minor lexical variants in device names referring to the same product—such as “medical image” vs “imaging”—were standardized to a single, consistent terminology. For the analysis of market concentration, “manufacturer” referred to the ultimate parent corporate entity. AIMDs approved under the legal names of subsidiaries belonging to the same corporate group were aggregated under the parent company’s name. A customized extraction form was piloted on 10 AIMDs and then applied independently by 2 reviewers (LZ and JY). Any disagreements were resolved through discussion until consensus was reached.

### Statistical Analysis

Descriptive statistics were used to summarize the extracted variables and assess temporal trends. We calculated the compound annual growth rate for annual approvals from 2020 to 2024 and applied the Mann-Kendall trend test to assess the significance of the growth trend. For AIMDs that used multiple data sources, each modality was counted separately in the subsequent analysis of data source distribution. Associations between categorical variables (approval year, medical specialty, and manufacturer’s city) and AIMD risk class were evaluated. The Fisher exact test was used for analyses involving variables with low expected cell counts (approval year and medical specialty), whereas the Pearson chi-square test was used for other comparisons. All tests were 2 sided, with *P*<.05 indicating statistical significance. Analyses were conducted using SPSS Statistics for Windows (version 27.0; IBM Corp).

### Ethical Considerations

This study was a secondary analysis of publicly available regulatory records retrieved from a database. No data from human participants or identifiable personal information were used, and no primary data collection was conducted. The data are anonymized and publicly accessible. In accordance with the institutional policy of China Pharmaceutical University, ethics board approval was not required for this study. This study complies with China’s Data Security Law [22] and Personal Information Protection Law [23].

## Results

### Overview of AIMD Approvals in China

**Figure 1.**
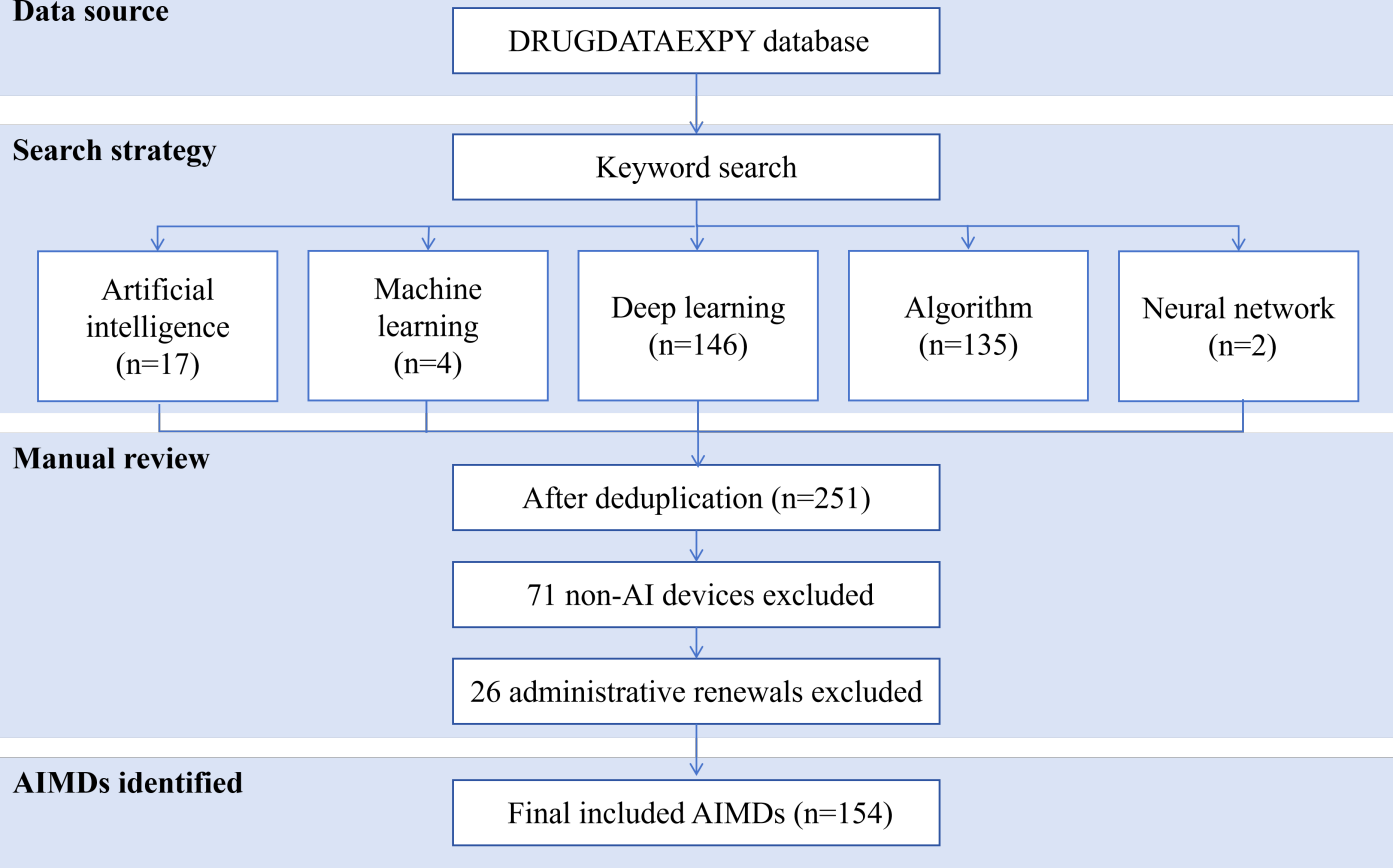
Inclusion flowchart for approved artificial intelligence (AI)–based medical devices (AIMDs) in China (2020‐2025).

**Figure 2.**
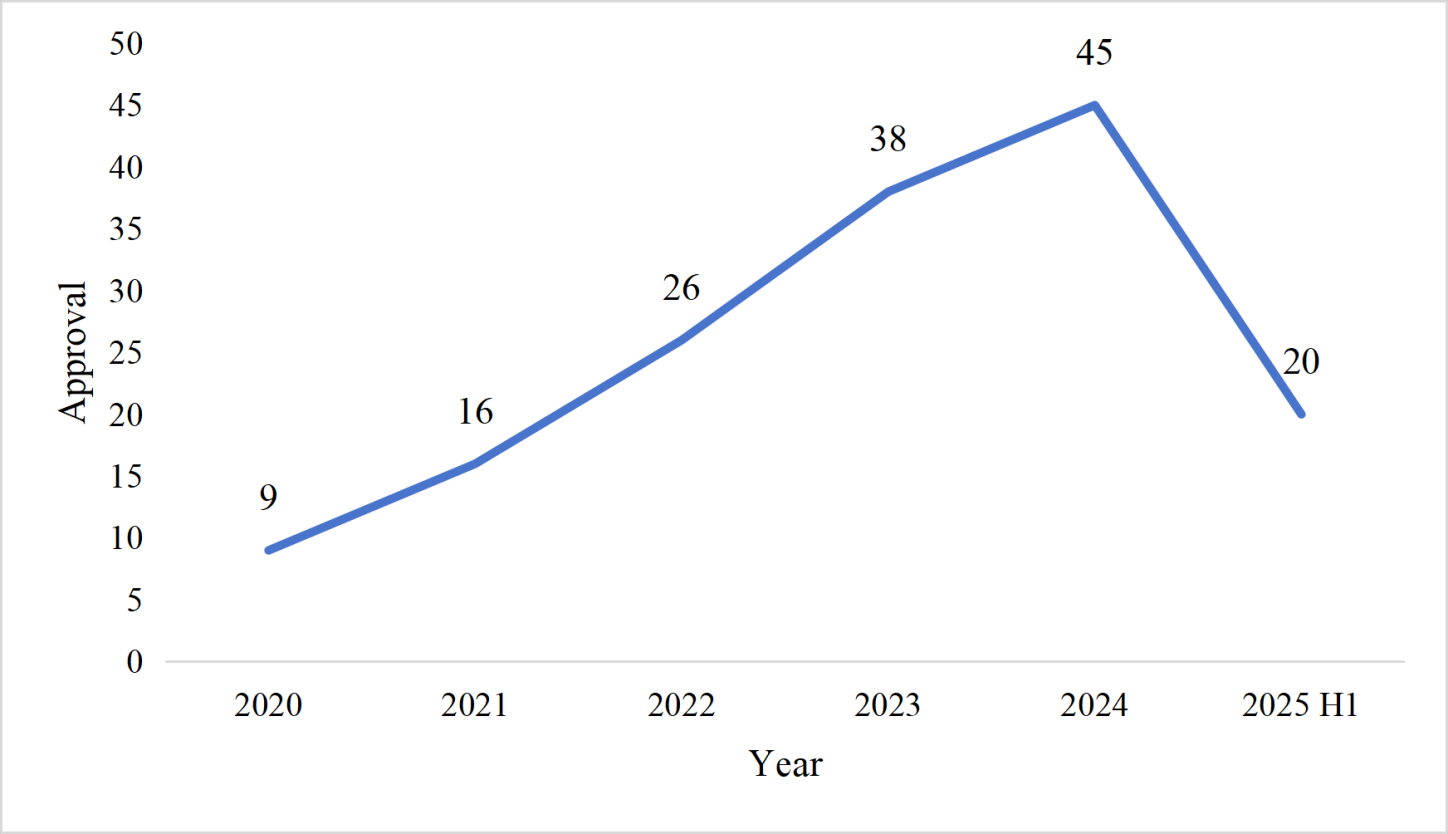
Trends in annual approvals of artificial intelligence–based medical devices in China (2020-2025; n=154). H1: first half of the year.

As of June 30, 2025, a total of 154 AIMDs had been approved in China since the initial authorization in 2020. Figure 1 shows the inclusion flowchart for AIMDs approved in China. All devices were intended for use by health care professionals, with none designed for direct patient use. The annual approval numbers exhibited a clear upward trend from 2020 to 2024: 9 in 2020, a total of 16 in 2021, a total of 26 in 2022, a total of 38 in 2023, and 45 in 2024. From 2020 to 2024, the compound annual growth rate was 49.53%, and the trend test confirmed a statistically significant increasing trend in annual approvals (*P*<.001). Data for the first half of 2025 show 20 approvals, indicating that the trend may be entering a phase of moderation or plateau (Figure 2). Detailed information on these AIMDs sorted by approval date is provided in Multimedia Appendix 2.

Among the 154 AIMD approvals, 15 device types had at least 3 approvals (Table 1). Pulmonary nodule CT image auxiliary detection software ranked first with 14 (9.1%) approvals. It was followed by coronary artery CT angiography image vessel stenosis auxiliary assessment software with 8 (5.2%) approvals. Coronary artery CT fractional flow reserve calculation software and intracranial hemorrhage CT image auxiliary triage software tied for third, each with 7 (4.5%) approvals. Diabetic retinopathy fundus image auxiliary diagnosis software and pneumonia CT image auxiliary triage and assessment software each had 6 (3.9%) approvals. Pediatric hand x-ray image auxiliary bone age assessment software had 4 (2.6%) approvals. A total of 8 additional products each obtained 3 (1.9%) approvals, spanning various applications from CT image processing and electrocardiogram analysis to specialized endoscopic, angiographic, and x-ray–based diagnostic tools.

**Table 1.** Artificial intelligence–based medical devices with ≥3 approvals in China (2020‐2025; n=154).

Device description	Approvals, n (%)
Pulmonary nodule CT^a^ image auxiliary detection software	14 (9.1)
Coronary artery CT angiography image vessel stenosis auxiliary assessment software	8 (5.2)
Coronary artery CT fractional flow reserve calculation software	7 (4.5)
Intracranial hemorrhage CT image auxiliary triage software	7 (4.5)
Diabetic retinopathy fundus image auxiliary diagnosis software	6 (3.9)
Pneumonia CT image auxiliary triage and assessment software	6 (3.9)
Pediatric hand x-ray image auxiliary bone age assessment software	4 (2.6)
CT image processing software	3 (1.9)
Dynamic electrocardiogram analysis software	3 (1.9)
Electrocardiogram analysis software	3 (1.9)
Electronic lower gastrointestinal endoscopy image auxiliary detection software for intestinal polyps	3 (1.9)
Head and neck CT angiography image auxiliary assessment software	3 (1.9)
Breast x-ray image auxiliary detection software	3 (1.9)
Medical image processing software	3 (1.9)
Rib fracture CT image auxiliary detection software	3 (1.9)

a CT: computed tomography.

### Data Source of AIMDs

As shown in Table 2, CT was the predominant data source for these AIMDs, identified in 62.3% (96/154) of approvals. This markedly exceeded other data sources, including x-ray (18/154, 11.7%), magnetic resonance imaging (12/154, 7.8%), endoscopy (9/154, 5.8%), electrocardiography (9/154, 5.8%), and fundus photography (9/154, 5.8%).

**Table 2.** Data sources used in approved artificial intelligence–based medical devices in China (2020‐2025). Devices using multiple data sources were counted under each relevant modality. Therefore, the sum of counts exceeds the total number of devices (n=154).

Data source	Devices, n (%)
CT^a^	96 (62.3)
X-ray	18 (11.7)
MRI^b^	12 (7.8)
Electrocardiogram	9 (5.8)
Endoscopy	9 (5.8)
Fundus photography	9 (5.8)
Microscopic images	6 (3.9)
Ultrasound	3 (1.9)
Polysomnography	1 (0.6)

a CT: computed tomography.

b MRI: magnetic resonance imaging.

### Risk Class and Correlation Analysis

**Figure 3.**
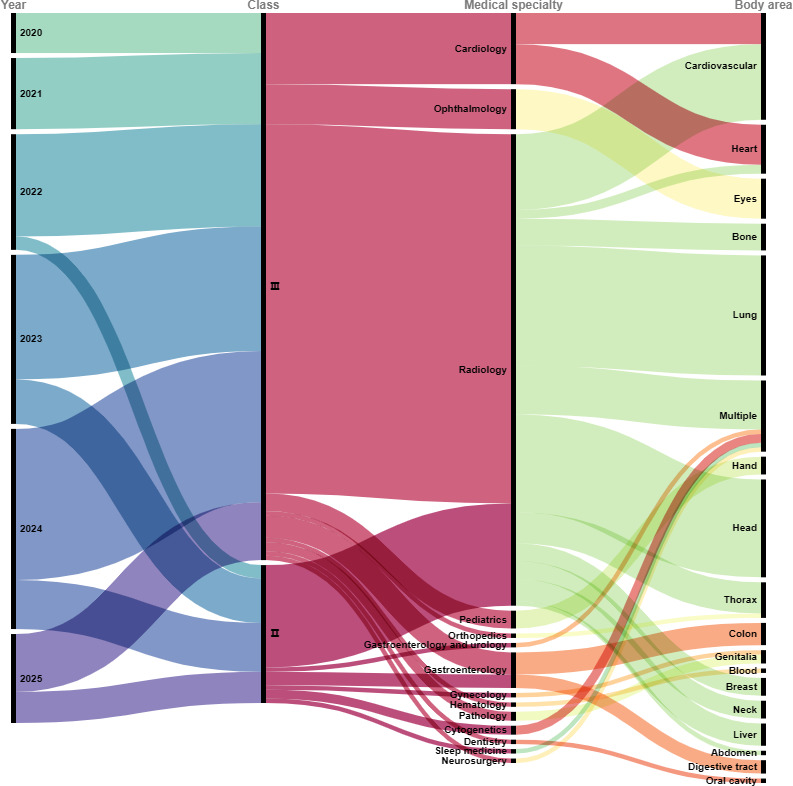
Sankey diagram of approved artificial intelligence–based medical devices in China (2020-2025).

The vast majority of AIMDs (123/154, 79.9%) were categorized as class III, with 20.1% (31/154) categorized as class II (Figure 3). Significant associations were found between risk class and 3 key variables: the year of approval (*P*=.03), the manufacturer’s city (*P*=.03), and the medical specialty (*P*=.004). In 2020 and 2021, only class III AIMDs were approved. After 2022, the share of class II AIMDs rose each year, from 11.5% (3/26) in 2022 to 35% (7/20) in 2025. The proportion of class III AIMDs was markedly higher in Hangzhou (16/16, 100%) and Beijing (47/54, 87%) than in other cities. The distribution of risk classes varied substantially across medical specialties. Ophthalmology and cardiology exhibited a 100% (16/16) class III rate, whereas 21.7% (23/106) of AIMDs in radiology were class II.

### Review Pathway and Algorithm

Of the 154 AIMDs, 23 (14.9%) gained market access through national-level special review pathways, which were exclusively for class III devices. Specifically, of the 123 class III devices, 19 (15.4%) were approved via innovation review, 2 (1.6%) were approved via priority review, and 2 (1.6%) were approved via emergency approval, constituting a combined 18.7% (23/123) of all class III approvals. The remaining 85.1% (131/154) of the devices followed the standard review process. Of the 154 AIMDs, 143 (92.9%) used deep learning architectures; 3 (1.9%) used classic ML methods; and for the remaining 8 (5.2%) devices, the algorithmic approach was not disclosed in the available registration materials.

### Clinical Evaluation Pathways

Analysis of the clinical evaluation pathways for the 154 AIMDs (Table 3) showed that most (n=118, 76.6%) submitted clinical trial reports as their primary evidence. The remaining devices were approved either through an exemption from clinical evaluation (n=23, 14.9%) or via a clinical comparison with similar products already available on the market (n=13, 8.4%).

Of the 123 class III devices, 116 (94.3%) used clinical trials, whereas clinical comparison with similar products and clinical exemption were less common (n=5, 4.1% and n=2, 1.6% of devices, respectively). In contrast, among the 31 class II devices, clinical exemption was the most frequent pathway (n=21, 67.7%), followed by clinical comparison with similar products (n=8, 25.8%) and clinical trials (n=2, 6.5%).

**Table 3.** Clinical evaluation pathways for approved artificial intelligence–based medical devices in China (2020‐2025; n=154).

	Total, n (%)	Class III devices (n=123), n (%)	Class II devices (n=31), n (%)
Clinical trial	118 (76.6)	116 (94.3)	2 (6.5)
Exemption from clinical evaluation	23 (14.9)	2 (1.6)	21 (67.7)
Clinical comparison with similar products	13 (8.4)	5 (4.1)	8 (25.8)

### Medical Specialty and Body Area

The 154 AIMDs spanned 14 medical specialties. Radiology dominated with 68.8% (n=106) of approvals, followed by cardiology (n=16, 10.4%), ophthalmology (n=9, 5.8%), and gastroenterology (n=9, 5.8%). The remaining AIMDs covered pediatrics, pathology, cytogenetics, sleep medicine, orthopedics, neurosurgery, hematology, gynecology, urology, and dentistry (Table 4).

The most common body areas for these AIMDs were the lung (27/154, 17.5%), the cardiovascular system (24/154, 15.6%), and the head (22/154, 14.3%), with the heart accounting for 7.1% (11/154). Additionally, 10.4% (16/154) of the AIMDs were used across multiple body areas (Table 5).

**Table 4.** Distribution of artificial intelligence–based medical devices by medical specialty in China (2020‐2025; n=154).

Medical specialty	Devices, n (%)
Radiology	106 (68.8)
Cardiology	16 (10.4)
Ophthalmology	9 (5.8)
Gastroenterology	9 (5.8)
Pediatrics	4 (2.6)
Others	10 (6.5)

**Table 5.** Distribution of artificial intelligence–based medical devices by target body area in China (2020‐2025; n=154).

Body area	Devices, n (%)
Lung	27 (17.5)
Cardiovascular system	24 (15.6)
Head	22 (14.3)
Multiple	16 (10.4)
Heart	11 (7.1)
Eyes	9 (5.8)
Thorax	8 (5.2)
Bone	6 (3.9)
Liver	5 (3.2)
Colon	5 (3.2)
Neck	4 (2.6)
Hand	4 (2.6)
Breast	4 (2.6)
Others	9 (5.8)

### Manufacturer and Geographic Distribution

We identified 67 manufacturers in total, with a high degree of market concentration. The top 4 companies alone produced 38.3% (59/154) of the AIMDs: Yukun and United Imaging led with 11% (17/154) of the devices each, followed by Infervision with 9.1% (14/154) and Deepwise with 7.1% (11/154). In contrast, 48 manufacturers had only 1 or 2 products each. Manufacturers with more than 2 approved AIMDs are shown in Table 6.

Geographically, of the 154 approved AIMDs, 54 (35.1%) were from manufacturers headquartered in Beijing, 32 (20.8%) were from Shanghai, 18 (11.7%) were from Shenzhen, and 16 (10.4%) were from Hangzhou (Table 7). Thus, these 4 cities accounted for 77.9% (n=120) of all the approved AIMDs. Beijing was home to Yukun and Infervision, Shanghai was home to United Imaging, and Hangzhou was home to Deepwise, which were precisely the top 4 companies listed above. Shenzhen hosted a broader ecosystem, including Carewell, Tencent Smart Healthcare, RuiXin, and Edan Instruments, but none brought more than 10 AIMDs to market.

**Table 6.** Manufacturers with ≥2 approved artificial intelligence–based medical devices in China (2020‐2025; n=154).

Manufacturer	Devices, n (%)
Yukun	17 (11.0)
United Imaging	17 (11.0)
Infervision	14 (9.1)
Deepwise	11 (7.1)
Yizhun	5 (3.2)
SenseTime	4 (2.6)
Carewell	4 (2.6)
Tongxin	3 (1.9)
Tencent Smart Healthcare	3 (1.9)
Siemens	3 (1.9)
RuiXin	3 (1.9)
Neusoft	3 (1.9)
Longwood Valley Medical	3 (1.9)
InnoVision	3 (1.9)
Huiyi Huiying	3 (1.9)
Edan Instruments	3 (1.9)
YiTu	2 (1.3)
Visionary Intelligence	2 (1.3)
Suzhou Volexcloud	2 (1.3)
Shukun	2 (1.3)
Keya Medical	2 (1.3)
Shenzhen Dr. Brain Technologies	2 (1.3)
BioMind	2 (1.3)

**Table 7.** Cities of manufacturers of approved artificial intelligence–based medical devices in China (2020‐2025; n=154).

Cities	Devices, n (%)
Beijing	54 (35.1)
Shanghai	32 (20.8)
Shenzhen	18 (11.7)
Hangzhou	16 (10.4)
Suzhou	4 (2.6)
Xiamen	4 (2.6)
Lishui	4 (2.6)
Shenyang	3 (1.9)
Kunming	3 (1.9)
Changsha	2 (1.3)
Wuhan	2 (1.3)
Guangzhou	2 (1.3)
Chengdu	2 (1.3)
Zhengzhou	1 (0.6)
Wuxi	1 (0.6)
Nanning	1 (0.6)
Nanjing	1 (0.6)
Nanchang	1 (0.6)
Longyan	1 (0.6)
Hefei	1 (0.6)
Guiyang	1 (0.6)

## Discussion

### Principal Findings

This study provided an overview of AIMDs in China up to June 2025. A total of 154 AIMDs were approved, and annual approvals showed an upward trend. Most of these AIMDs (123/154, 79.9%) were class III and relied on deep learning. They were concentrated in radiology, particularly CT-based; produced by a small number of manufacturers; and located in a few cities.

China did not approve its first AIMD until 2020, later than both the United States and Europe, where approvals for AIMDs occurred as early as 2015 [10] despite variations in the definitions of AIMDs. The number of AIMD approvals in China remains lower than that in the United States and Europe. However, we have observed a rapid increase in AIMD approvals in China, reflecting technological advancements and a supportive regulatory environment.

Our analysis, extending the timeline to mid-2025, built on and updated the foundational work of Liu et al [14]. While confirming the continued dominance of radiology and class III devices previously reported, our data revealed new dynamics. Specifically, we observed a marked increase in the absolute number of approvals and a significant shift with the emergence of class II AIMDs after 2022—a trend that was not fully evident in the earlier data. Furthermore, the sustained market concentration among top manufacturers and in key geographic hubs suggests that these early structural characteristics consolidated rather than dispersed as the market grew. These comparisons highlight the rapid evolution of this field and underscore the value of longitudinal tracking to inform regulatory and market strategies. It should be noted that our counts for 2021 to 2022 were slightly higher than those reported by Liu et al [14]. We identified additional AIMDs, including image processing software and CT fractional flow reserve calculation software. A primary reason for this discrepancy is a fundamental difference in data scope. Our dataset encompassed both centrally approved class III devices and provincially approved class II devices, whereas Liu et al [14] primarily focused on class III approvals granted by the NMPA. Additionally, differences can be attributed to distinct search strategies: our approach incorporated AI-related keywords—such as “deep learning” and “algorithm”—within the “structural composition” and “intended use” fields, whereas their search relied on device-naming conventions, using terms such as “computer-assisted detection” and “computer-assisted triage.”

A prime example of an AIMD category with significant clinical penetration in China is pulmonary nodule CT image auxiliary detection software. This category had 14 approved variants from multiple manufacturers, making it the most widespread AIMD type. According to manufacturer data, United Imaging has deployed its solution in over 2000 hospitals nationwide. Their clinical evaluations indicate that the software can increase the detection rate of pulmonary nodules by approximately 32% and improve radiologists’ reading efficiency by approximately 26% compared with conventional methods [24].

Regarding risk classification, most Chinese AIMDs are designated as class III, whereas all US AIMDs are cleared as class II [25] and Europe has only approximately 1% of AIMDs in class III [11]. The large share of class III in China suggests that many AIMDs were treated as high risk and required the strictest review. From 2020 to 2021, only class III AIMDs were observed; from 2022 onward, this pattern shifted, with a steady influx of class II products. The observed change can be attributed to the *Principles for the Classification Defining of AI-Based Medical Software Products* issued by the NMPA in 2021 [26], which applies to both software as a medical device and SiMD. For SiMD, the assigned risk class applies to the product as a whole, reflecting a holistic regulatory assessment of the AI functionality’s clinical role within the device. This guideline allowed AIMDs with mature algorithms that provided only non–real-time assistance—such as measurement, marking, or workflow optimization—to be placed in class II. Previously, any software bearing AI technology had been categorized as class III. The significant association between risk classification and the manufacturer’s city likely reflects regional policy support and the clustering of expertise in cities such as Beijing and Hangzhou, where high-risk AIMD development appears concentrated.

Our analysis further revealed that the vast majority of approved AIMDs were supported by premarket clinical evidence through clinical trials or clinical comparison with similar products. Exemption from clinical evaluation was granted for only 23 AIMDs, 21 (91.3%) of which were class II and predominantly consisted of image processing software. This pattern reflects the practical implementation of a risk-based regulatory framework: higher-risk AIMDs generally necessitate more robust premarket clinical validation, while pathways such as exemption or comparison are more frequently applied to lower-risk AIMDs to facilitate efficient market access. Nevertheless, reliance on exemptions and comparative assessments for lower-risk approvals raises concerns about the sufficiency and transparency of premarket evidence. To strengthen the regulatory framework, we recommend clarifying exemption eligibility criteria and tightening the evidentiary standards for demonstrating equivalence.

We did not identify any safety-related recalls among the 154 AIMDs approved as of June 2025. A recent study of Food and Drug Administration–authorized AI and ML devices found that 5.2% were linked to adverse events and 5.8% were recalled, primarily due to software issues [27]. The lack of similar public reports in China may stem from its later market start and differences in data transparency. Accordingly, establishing a robust postmarket surveillance system is essential to detect risks arising from algorithm or workflow changes, and it requires commitments from regulators and manufacturers to ensure the ongoing safety of AIMDs.

Radiology dominated China’s AIMD landscape, with an emphasis on CT-based applications, reflecting alignment with the nation’s health care needs and its existing technological infrastructure. This focus is evident in the concentration of devices targeting pulmonary nodules and cardiovascular conditions, both representing substantial disease burden. Pulmonary nodules are clinically significant as early indicators of lung cancer, which remains the most common cancer type and the leading cause of cancer death in China [28]. Similarly, cardiovascular diseases rank as the leading cause of overall mortality, accounting for approximately 2 out of every 5 deaths [29].

The high proportion of radiology-related AIMDs approved in China aligns with the trend observed in the United States [25]. Medical imaging modalities such as CT, magnetic resonance imaging, and x-ray generate structured, digital data well suited for AI applications. Among these modalities, CT images have formed the basis for most AIMDs developed in China. The widespread availability of CT scanners in Chinese health care facilities, together with relatively uniform imaging protocols, has accelerated the growth of AI diagnostic software based on CT.

Market concentration emerged as another notable finding, with a limited number of manufacturers accounting for a large share of approved AIMDs. Notably, these dominant manufacturers were primarily headquartered in major cities such as Beijing, Shanghai, and Hangzhou. This market dominance appears to stem from the convergence of several reinforcing factors. These include the concentration of technical and regulatory expertise, preferential access to high-quality medical data through partnerships with leading hospitals, and the availability of substantial venture capital in these regions [30,31].

The observed characteristics of China’s AIMD market, specifically its pronounced concentration in clinical specialties and geographic regions, were interpreted through the lens of innovation diffusion theory. First, radiology dominated the field, especially CT-based applications, illustrating innovations with high compatibility through seamless integration into digital imaging workflows and a clear relative advantage in improving efficiency during high-volume screenings, which promotes rapid early adoption. Second, the geographic concentration of approvals in Beijing, Shanghai, Shenzhen, and Hangzhou reflects the social system effect. These megacities concentrate critical resources for diffusion, such as policy incentives, venture capital, and technical talent, forming powerful hubs that accelerate local development and initial market penetration of AIMDs.

The observed approval trends could not be fully understood without considering China’s evolving regulatory framework. Our data showed sustained growth consistent with the aims of recent NMPA reforms. Notably, the 2025 measures to optimize full life cycle supervision and support the innovative development of advanced medical devices explicitly aimed to accelerate reviews of advanced devices, optimize premarket processes, and strengthen postmarket surveillance. This policy shift provided essential context for interpreting market dynamics and indicated a regulatory environment oriented toward facilitating efficient innovation while ensuring safety throughout a product’s life cycle. Furthermore, the emphasis on fostering high-level industrial clusters offered a lens to interpret the pronounced geographic concentration of approvals observed in this study. The synergy between concentrated innovation resources in hubs such as Beijing and Shanghai and proactive regulatory support appears to be a defining feature of China’s AIMD ecosystem.

### Limitations

This study has several limitations. First, the identification of an AIMD depended on the explicit mention of AI techniques in its functional components or intended use. Devices that did not explicitly state the use of AI might have been missed despite cross-checking ambiguous cases with review reports and websites. Second, classifying medical specialties was challenging because many devices spanned multiple clinical domains. We assigned a single primary specialty, which may underrepresent secondary applications. Third, although we standardized device names by merging minor variations that referred to the same product, some inconsistencies may persist, underscoring the need for unified naming conventions.

### Conclusions

This analysis indicated that AIMDs in China are experiencing rapid growth. The regulatory landscape is dominated by radiology applications, with CT-based solutions for pulmonary nodules and cardiovascular conditions especially prominent. There is also a high degree of market concentration among leading manufacturers.

## Supplementary material

10.2196/85538 Multimedia Appendix 1 Search strategy and process for identifying artificial intelligence–based medical devices.

10.2196/85538 Multimedia Appendix 2 Detailed information of approved artificial intelligence–based medical devices in China.

## Abbreviations

AI: artificial intelligence
AIMD: artificial intelligence–based medical device
CT: computed tomography
ML: machine learning
NMPA: National Medical Products Administration
SiMD: software in a medical device
