# Supplementary material for: Approval of AI-Based Medical Devices in China From 2020 to 2025: Retrospective Analysis
Source: JMIR Med Inform. 2026 Mar 18;14:e85538. doi: 10.2196/85538 (PMC12998604; doi:10.2196/85538)
Supplement: Multimedia Appendix 1 [file medinform-v14-e85538-s001.docx]

**Multimedia Appendix 1.** Search strategy and process for identifying AI-based medical devices.

| **Component** | **Item** | **Specification** |
| --- | --- | --- |
| 1. Data Source | Database | Drugdataexpy (https://db.yaozh.com/) |
|  | Time frame | Inception to June 30, 2025. |
| 2. Search Strategy | Search fields | Structural composition and intended use |
|  | Search terms (Chinese) | Artificial intelligence, machine learning, deep learning, neural network, algorithm |
| 3. Record Processing | Deduplication | After pooling records retrieved from separate single-keyword searches, duplicates were removed by identifying and merging multiple entries. |
|  | Exclusion - non-AI devices | Manually reviewed full text to exclude medical devices that core functionality did not rely on AI algorithms |
|  | Exclusion - administrative renewals | Excluded renewal registrations or changes that did not involve the addition of AI functions (e.g. manufacturing address) |
|  | Final included number | **N = 154** |
