# Supplementary material for: Approval of AI-Based Medical Devices in China From 2020 to 2025: Retrospective Analysis
Source: JMIR Med Inform. 2026 Mar 18;14:e85538. doi: 10.2196/85538 (PMC12998604; doi:10.2196/85538)
Supplement: Multimedia Appendix 2 [file medinform-v14-e85538-s002.docx]

**Multimedia Appendix 2.** Detailed information of approved AI-based medical devices in China.

| **Authority** | **Registration Number** | **Device Name** | **Data Source** | **Medical Specialty** | **Body Area** | **Manufacturer** | **City of Manufacturer** | **Approval Date** | **Class** | **Algorithm** | **Review Procedure** |
| --- | --- | --- | --- | --- | --- | --- | --- | --- | --- | --- | --- |
| NMPA | 20203210035 | Coronary Artery CT Fractional Flow Reserve (FFR) Calculation Software | CT | Cardiology | Cardiovascular | Keya Medical | Beijing | 2020/1/14 | Ⅲ | Deep learning | Innovation |
| NMPA | 20203210080 | Electrocardiogram (ECG) Analysis Software | ECG | Cardiology | Heart | Carewell | Shenzhen | 2020/1/22 | Ⅲ | Deep learning | Standard |
| NMPA | 20203070658 | Electrocardiograph | ECG | Cardiology | Heart | Carewell | Shenzhen | 2020/7/22 | Ⅲ | Deep learning | Standard |
| NMPA | 20203210686 | Diabetic Retinopathy Fundus Image Auxiliary Diagnosis Software | Fundus photography | Ophthalmology | Eyes | Shanghai Airdoc | Shanghai | 2020/8/7 | Ⅲ | Deep learning | Innovation |
| NMPA | 20203210687 | Diabetic Retinopathy Fundus Image Auxiliary Diagnosis Software | Fundus photography | Ophthalmology | Eyes | Shenzhen Silicon Intelligence | Shenzhen | 2020/8/7 | Ⅲ | Deep learning | Innovation |
| NMPA | 20203210844 | Coronary Artery CT Angiography Image Vessel Stenosis Auxiliary Triage Software | CT | Radiology | Cardiovascular | Yukun | Beijing | 2020/11/3 | Ⅲ | Deep learning | Innovation |
| NMPA | 20203210862 | Fracture CT Image Auxiliary Detection Software | CT | Radiology | Bone | Huiyihuiying (Xiamen) | Xiamen | 2020/11/9 | Ⅲ | Deep learning | Standard |
| NMPA | 20203210920 | Pulmonary Nodule CT Image Auxiliary Detection Software | CT | Radiology | Lung | Infervision | Beijing | 2020/11/30 | Ⅲ | Deep learning | Innovation |
| NMPA | 20203210839 | Pulmonary Nodule CT Image Auxiliary Detection Software | CT | Radiology | Lung | Deepwise (Hangzhou) | Hangzhou | 2020/12/11 | Ⅲ | Deep learning | Standard |
| NMPA | 20213210032 | Radiation Therapy Contouring Software | CT | Radiology | Multiple | United Imaging (Shanghai) | Shanghai | 2021/1/15 | Ⅲ | Deep learning | Standard |
| NMPA | 20213070059 | Multi-Channel Electrocardiograph | ECG | Cardiology | Heart | Carewell | Shenzhen | 2021/1/26 | Ⅲ | Deep learning | Standard |
| NMPA | 20213210177 | Pediatric Hand X-ray Image Auxiliary Bone Age Assessment Software | X-ray | Pediatrics | Hand | YiTu | Hangzhou | 2021/3/18 | Ⅲ | Deep learning | Priority |
| NMPA | 20213210211 | Pneumonia CT Image Auxiliary Triage and Assessment Software | CT | Radiology | Lung | Infervision | Beijing | 2021/3/26 | Ⅲ | Deep learning | Emergency Review |
| NMPA | 20213210210 | Pneumonia CT Image Auxiliary Triage and Assessment Software | CT | Radiology | Lung | Deepwise (Hangzhou) | Hangzhou | 2021/3/26 | Ⅲ | Deep learning | Emergency Review |
| NMPA | 20213210270 | Coronary Artery CT Fractional Flow Reserve (FFR) Calculation Software | CT | Cardiology | Cardiovascular | RuiXin | Shenzhen | 2021/4/14 | Ⅲ | Deep learning | Innovation |
| NMPA | 20213210308 | Fracture X-ray Image Auxiliary Detection Software | X-ray | Radiology | Bone | United Imaging (Shanghai) | Shanghai | 2021/4/28 | Ⅲ | Deep learning | Standard |
| NMPA | 20213210422 | Diabetic Retinopathy Fundus Image Auxiliary Diagnosis Software | Fundus photography | Ophthalmology | Eyes | Visionary Intelligence | Beijing | 2021/6/8 | Ⅲ | Undisclosed | Standard |
| NMPA | 20213210471 | Pulmonary Nodule CT Image Auxiliary Detection Software | CT | Radiology | Lung | United Imaging (Shanghai) | Shanghai | 2021/6/24 | Ⅲ | Deep learning | Standard |
| NMPA | 20213210574 | Coronary Artery CT Fractional Flow Reserve (FFR) Calculation Software | CT | Cardiology | Cardiovascular | Heart Century | Beijing | 2021/7/29 | Ⅲ | Deep learning | Innovation |
| NMPA | 20213210607 | Pneumonia CT Image Auxiliary Triage and Assessment Software | CT | Radiology | Lung | United Imaging (Shanghai) | Shanghai | 2021/8/6 | Ⅲ | Deep learning | Standard |
| NMPA | 20213210612 | Pneumonia CT Image Auxiliary Triage and Assessment Software | CT | Radiology | Lung | Tencent Healthcare | Shenzhen | 2021/8/16 | Ⅲ | Deep learning | Standard |
| NMPA | 20213210911 | Pneumonia CT Image Auxiliary Triage and Assessment Software | CT | Radiology | Lung | BioMind | Beijing | 2021/11/12 | Ⅲ | Deep learning | Standard |
| NMPA | 20213210968 | Pulmonary Nodule CT Image Auxiliary Triage Software | CT | Radiology | Lung | Suzhou Volexcloud | Suzhou | 2021/11/18 | Ⅲ | Deep learning | Standard |
| NMPA | 20213211007 | Pneumonia CT Image Auxiliary Triage and Assessment Software | CT | Radiology | Lung | Yukun | Beijing | 2021/12/1 | Ⅲ | Deep learning | Standard |
| NMPA | 20213211094 | Pulmonary Nodule CT Image Auxiliary Detection Software | CT | Radiology | Lung | YiTu | Hangzhou | 2021/12/23 | Ⅲ | Deep learning | Standard |
| NMPA | 20223210295 | Pediatric Hand X-ray Image Auxiliary Bone Age Assessment Software | X-ray | Pediatrics | Hand | Deepwise (Hangzhou) | Hangzhou | 2022/3/2 | Ⅲ | Deep learning | Standard |
| NMPA | 20223210309 | Intracranial Hemorrhage CT Image Auxiliary Triage Software | CT | Radiology | Head | United Imaging (Shanghai) | Shanghai | 2022/3/9 | Ⅲ | Deep learning | Innovation |
| NMPA | 20223210445 | Diabetic Retinopathy Fundus Image Auxiliary Diagnosis Software | Fundus photography | Ophthalmology | Eyes | Fujian Wedoctor | Longyan | 2022/4/6 | Ⅲ | Deep learning | Standard |
| NMPA | 20223210482 | Head and Neck CT Angiography Image Auxiliary Assessment Software | CT | Radiology | Head | Yukun | Beijing | 2022/4/12 | Ⅲ | Deep learning | Standard |
| NMPA | 20223210572 | Chest Fracture CT Image Auxiliary Triage Software | CT | Orthopedics | Thorax | Infervision | Beijing | 2022/4/29 | Ⅲ | Deep learning | Standard |
| NMPA | 20223210575 | Pulmonary Nodule CT Image Auxiliary Detection Software | CT | Radiology | Lung | Huiyihuiying (Beijing) | Beijing | 2022/4/29 | Ⅲ | Deep learning | Standard |
| NMPA | 20223210570 | Pulmonary Nodule CT Image Auxiliary Detection Software | CT | Radiology | Lung | Yukun | Beijing | 2022/4/29 | Ⅲ | Deep learning | Standard |
| NMPA | 20223210625 | Pulmonary Nodule CT Image Auxiliary Detection Software | CT | Radiology | Lung | Fosun Aitrox | Shanghai | 2022/5/13 | Ⅲ | Deep learning | Standard |
| NMPA | 20223210687 | Pulmonary Nodule CT Image Auxiliary Detection Software | CT | Radiology | Lung | Yizhun | Nanning | 2022/5/26 | Ⅲ | Deep learning | Standard |
| NMPA | 20223210775 | Intracranial Hemorrhage CT Image Auxiliary Triage Software | CT | Radiology | Head | Infervision | Beijing | 2022/6/27 | Ⅲ | Deep learning | Standard |
| NMPA | 20223210896 | Diabetic Retinopathy Fundus Image Auxiliary Diagnosis Software | Fundus photography | Ophthalmology | Eyes | Suzhou Volexcloud | Suzhou | 2022/7/13 | Ⅲ | Deep learning | Standard |
| Henan | 20222210875 | Endoscopic Image Processing Auxiliary Examination Software | Endoscopy | Gastroenterology, Urology | Multiple | Xuanwei | Zhengzhou | 2022/7/15 | Ⅱ | Undisclosed | Standard |
| NMPA | 20223210981 | Electronic Colonoscopy Image Auxiliary Detection Software for Colorectal Polyps | Endoscopy | Gastroenterology | Colon | Wision AI | Chengdu | 2022/8/2 | Ⅲ | Deep learning | Innovation |
| NMPA | 20223211038 | Cerebral Ischemia Image Auxiliary Assessment Software | CT, MRI | Radiology | Head | Neusoft | Shenyang | 2022/8/4 | Ⅲ | Deep learning | Standard |
| NMPA | 20223211099 | Cardiovascular CT Image Auxiliary Assessment Software | CT | Radiology | Cardiovascular | Siemens | Shanghai | 2022/8/16 | Ⅲ | Deep learning | Standard |
| NMPA | 20223211102 | Diabetic Retinopathy Fundus Image Auxiliary Diagnosis Software | Fundus photography | Ophthalmology | Eyes | Zhen Health | Beijing | 2022/8/16 | Ⅲ | Deep learning | Standard |
| NMPA | 20223211081 | Fundus Image Auxiliary Diagnosis Software for Fundus Lesions | Fundus photography | Ophthalmology | Eyes | Kangfuzi | Beijing | 2022/8/16 | Ⅲ | Deep learning | Standard |
| NMPA | 20223210998 | Thoracic Spine CT Image Auxiliary Assessment Software | CT | Radiology | Thorax | Siemens | Shanghai | 2022/8/16 | Ⅲ | Deep learning | Standard |
| NMPA | 20223211140 | Fundus Image Auxiliary Diagnosis Software for Chronic Glaucomatous Optic Neuropathy | Fundus photography | Ophthalmology | Eyes | Tencent Healthcare | Shenzhen | 2022/8/31 | Ⅲ | Deep learning | Innovation |
| NMPA | 20223211142 | Dynamic Electrocardiogram (ECG) Analysis Software | ECG | Cardiology | Heart | Carewell | Shenzhen | 2022/9/1 | Ⅲ | Deep learning | Standard |
| Hunan | 20222211824 | Gynecological Microecology Auxiliary Analysis Software | Microscopic images | Gynecology | Genitalia | Weizhi | Changsha | 2022/9/29 | Ⅱ | Deep learning | Standard |
| NMPA | 20223211346 | Intracranial Aneurysm Surgery Planning Software | X-ray | Radiology | Head | Unistrong Technology | Beijing | 2022/10/11 | Ⅲ | Deep learning | Innovation |
| NMPA | 20223211374 | Pulmonary Tuberculosis X-ray Image Auxiliary Assessment Software | X-ray | Radiology | Lung | Jiufeng Healthcare | Nanchang | 2022/10/20 | Ⅲ | Deep learning | Priority |
| Fujian | 20222210119 | Bone Density CT Image Auxiliary Detection Software | CT | Radiology | Bone | United Imaging (Shanghai) | Shanghai | 2022/10/21 | Ⅱ | Deep learning | Standard |
| NMPA | 20223211426 | Electrocardiogram (ECG) Analysis Software | ECG | Cardiology | Heart | Nalong health | Xiamen | 2022/10/26 | Ⅲ | Deep learning | Standard |
| NMPA | 20223211473 | Peripheral Blood Cell Image White Blood Cell Auxiliary Recognition Software | Microscopic images | Hematology | Blood | Aimagine | Beijing | 2022/11/4 | Ⅲ | Deep learning | Standard |
| NMPA | 20233210006 | Breast X-ray Image Auxiliary Detection Software | X-ray | Radiology | Breast | Deepwise (Hangzhou) | Hangzhou | 2023/1/5 | Ⅲ | Deep learning | Standard |
| NMPA | 20233210146 | Coronary Artery CT Fractional Flow Reserve (FFR) Calculation Software | CT | Cardiology | Cardiovascular | Yukun | Beijing | 2023/2/3 | Ⅲ | Deep learning | Standard |
| NMPA | 20233210186 | Coronary Artery CT Angiography Image Vessel Stenosis Auxiliary Assessment Software | CT | Radiology | Cardiovascular | Yukun | Beijing | 2023/2/22 | Ⅲ | Deep learning | Standard |
| NMPA | 20233210216 | Coronary Artery CT Angiography Image Vessel Stenosis Auxiliary Assessment Software | CT | Radiology | Cardiovascular | Deepwise (Hangzhou) | Hangzhou | 2023/2/27 | Ⅲ | Deep learning | Standard |
| NMPA | 20233210249 | Electrocardiogram (ECG) Atrial Fibrillation Analysis Software | ECG | Cardiology | Heart | Edan Instruments | Shenzhen | 2023/3/7 | Ⅲ | Deep learning | Standard |
| NMPA | 20233210272 | Cervical Cytology Digital Pathology Image Computer-Aided Analysis Software | Microscopic images | Pathology | Genitalia | 91360 Medical Technology | Nanjing | 2023/3/8 | Ⅲ | Deep learning | Standard |
| NMPA | 20233210450 | Coronary Artery CT Fractional Flow Reserve (FFR) Calculation Software | CT | Cardiology | Cardiovascular | Pulse Medical | Shanghai | 2023/4/4 | Ⅲ | Deep learning | Innovation |
| NMPA | 20233210405 | Intracranial Hemorrhage CT Image Auxiliary Triage Software | CT | Radiology | Head | Yukun | Beijing | 2023/4/10 | Ⅲ | Deep learning | Standard |
| Guizhou | 20182210010* | Lung Digital Medical Image Processing Software | X-ray,CT | Radiology | Lung | Tiger Medic | Guiyang | 2023/4/10 | Ⅱ | Deep learning | Standard |
| NMPA | 20233210530 | Pulmonary Nodule CT Image Auxiliary Detection Software | CT | Radiology | Lung | Ifly Health | Hefei | 2023/4/23 | Ⅲ | Deep learning | Standard |
| NMPA | 20233210568 | Rib Fracture CT Image Auxiliary Detection Software | CT | Radiology | Thorax | Deepwise (Hangzhou) | Hangzhou | 2023/4/28 | Ⅲ | Deep learning | Standard |
| NMPA | 20233210629 | Electronic Lower Gastrointestinal Endoscopy Image Auxiliary Detection Software for Intestinal Polyps | Endoscopy | Gastroenterology | Colon | ENDOANGEL Medical Technology | Wuhan | 2023/5/12 | Ⅲ | Deep learning | Innovation |
| NMPA | 20233210695 | Rib Fracture CT Image Auxiliary Detection Software | CT | Radiology | Thorax | Yukun | Beijing | 2023/5/31 | Ⅲ | Deep learning | Standard |
| NMPA | 20233210707 | Electronic Endoscopy Image Auxiliary Detection Software for Colonic Polyps | Endoscopy | Gastroenterology | Colon | Tencent Healthcare | Shenzhen | 2023/6/1 | Ⅲ | Deep learning | Innovation |
| Liaoning | 20232210056 | Coronary Artery CT Angiography Image Vessel Auxiliary Processing Software | CT | Radiology | Cardiovascular | Neusoft | Shenyang | 2023/7/4 | Ⅱ | Deep learning | Standard |
| Yunnan | 20232210030 | CT Image Processing Software | CT | Radiology | Multiple | Tongxin | Kunming | 2023/7/4 | Ⅱ | Undisclosed | Standard |
| Yunnan | 20232210031 | DSA Image Processing Software | X-ray | Radiology | Multiple | Tongxin | Kunming | 2023/7/4 | Ⅱ | Undisclosed | Standard |
| Yunnan | 20232210032 | MR Image Processing Software | MRI | Radiology | Multiple | Tongxin | Kunming | 2023/7/10 | Ⅱ | Undisclosed | Standard |
| NMPA | 20233210974 | Breast X-ray Image Auxiliary Detection Software | X-ray | Radiology | Breast | Yizhun (Zhejiang) | Lishui | 2023/7/13 | Ⅲ | Deep learning | Standard |
| NMPA | 20233210976 | Ischemic Stroke CT Image Auxiliary Assessment Software | CT | Radiology | Head | Yukun | Beijing | 2023/7/13 | Ⅲ | Deep learning | Standard |
| Shanghai | 20232210198 | Lung CT Medical Image Processing Software | CT | Radiology | Lung | Shukun (Shanghai) | Shanghai | 2023/7/28 | Ⅱ | Deep learning | Standard |
| NMPA | 20233211066 | Coronary Artery CT Angiography Image Vessel Stenosis Auxiliary Assessment Software | CT | Radiology | Cardiovascular | United Imaging (Shanghai) | Shanghai | 2023/8/1 | Ⅲ | Deep learning | Standard |
| NMPA | 20233211114 | Coronary Artery CT Angiography Image Vessel Stenosis Auxiliary Assessment Software | CT | Radiology | Cardiovascular | Infervision | Beijing | 2023/8/4 | Ⅲ | Deep learning | Standard |
| Guangdong | 20232211491 | Great Vessel CT Image Processing Software | CT | Radiology | Cardiovascular | RuiXin | Shenzhen | 2023/9/12 | Ⅱ | Deep learning | Standard |
| NMPA | 20233211362 | Magnetic Resonance Image Auxiliary Assessment Software | MRI | Radiology | Head | Dr Brain | Shenzhen | 2023/9/19 | Ⅲ | Deep learning | Standard |
| NMPA | 20233211543 | Orthopedic Surgery Simulation Software | X-ray, CT | Radiology | Bone | Huiyihuiying (Beijing) | Beijing | 2023/10/23 | Ⅲ | Deep learning | Innovation |
| NMPA | 20233211548 | Head and Neck CT Angiography Image Auxiliary Assessment Software | CT | Radiology | Head | Deepwise (Hangzhou) | Hangzhou | 2023/10/25 | Ⅲ | Deep learning | Standard |
| Beijing | 20232210688 | CT Image Processing Software | CT | Radiology | Heart | Yukun | Beijing | 2023/10/30 | Ⅱ | Deep learning | Standard |
| NMPA | 20233211594 | Pulmonary Nodule CT Image Auxiliary Detection Software | CT | Radiology | Lung | United Imaging (Shanghai) | Shanghai | 2023/11/1 | Ⅲ | Deep learning | Standard |
| NMPA | 20233211648 | Electronic Lower Gastrointestinal Endoscopy Image Auxiliary Detection Software for Intestinal Polyps | Endoscopy | Gastroenterology | Colon | InnoVision (Xiamen) | Xiamen | 2023/11/8 | Ⅲ | Deep learning | Standard |
| NMPA | 20233211650 | Thyroid Nodule Ultrasound Image Auxiliary Diagnosis Software | Ultrasound | Radiology | Neck | Emetics Medical | Hangzhou | 2023/11/8 | Ⅲ | Deep learning | Standard |
| NMPA | 20233211732 | Intracranial Aneurysm CT Angiography Image Auxiliary Triage Software | CT | Radiology | Head | Yukun | Beijing | 2023/11/22 | Ⅲ | Deep learning | Standard |
| NMPA | 20233211737 | Pediatric Hand X-ray Image Auxiliary Bone Age Assessment Software | X-ray | Pediatrics | Hand | Yukun | Beijing | 2023/11/22 | Ⅲ | Deep learning | Standard |
| NMPA | 20233211772 | Focal Liver Lesion MR Image Auxiliary Triage Software | MRI | Radiology | Liver | Yukun | Beijing | 2023/11/28 | Ⅲ | Deep learning | Standard |
| NMPA | 20233211836 | Pulmonary Nodule CT Image Auxiliary Detection Software | CT | Radiology | Lung | Jianpei | Hangzhou | 2023/12/4 | Ⅲ | Deep learning | Standard |
| Shanghai | 20232210338 | CT Image Processing Software | CT | Radiology | Heart | United Imaging (Shanghai) | Shanghai | 2023/12/10 | Ⅱ | Undisclosed | Standard |
| Shanghai | 20232210342 | Coronary CT Image 3D Reconstruction Software | CT | Radiology | Cardiovascular | Sensetime | Shanghai | 2023/12/14 | Ⅱ | Deep learning | Standard |
| NMPA | 20233212016 | Chest CT Image Processing and Analysis Software | CT | Radiology | Thorax | Infervision | Beijing | 2023/12/25 | Ⅲ | Deep learning | Standard |
| NMPA | 20243210066 | Pulmonary Nodule CT Image Auxiliary Detection Software | CT | Radiology | Lung | Siemens | Shanghai | 2024/1/9 | Ⅲ | Deep learning | Standard |
| Shanghai | 20242210020 | Head and Neck Artery CT Image 3D Reconstruction Software | CT | Radiology | Head | Sensetime | Shanghai | 2024/1/16 | Ⅱ | Deep learning | Standard |
| NMPA | 20243210226 | Dynamic Electrocardiogram (ECG) Analysis Software | ECG | Cardiology | Heart | Thoth | Suzhou | 2024/1/31 | Ⅲ | Deep learning | Standard |
| NMPA | 20243210223 | Rib Fracture CT Image Auxiliary Detection Software | CT | Radiology | Thorax | Yizhun (Zhejiang) | Lishui | 2024/1/31 | Ⅲ | Deep learning | Standard |
| NMPA | 20243210396 | Intracranial Aneurysm CT Angiography Image Auxiliary Detection Software | CT | Radiology | Head | Deepwise (Hangzhou) | Hangzhou | 2024/3/1 | Ⅲ | Deep learning | Innovation |
| NMPA | 20243210509 | Liver and Kidney Surgery Planning Software | CT | Radiology | Abdomen | Infervision | Beijing | 2024/3/13 | Ⅲ | Deep learning | Standard |
| Shanghai | 20242210082 | Medical Image Processing Software | X-ray | Radiology | Multiple | Spinex | Shanghai | 2024/3/20 | Ⅱ | Deep learning | Standard |
| NMPA | 20243210597 | Coronary Artery CT Fractional Flow Reserve (FFR) Calculation Software | CT | Cardiology | Cardiovascular | Deepwise (Hangzhou) | Hangzhou | 2024/3/26 | Ⅲ | Deep learning | Standard |
| Guangdong | 20232210410 | Gastrointestinal Tract Image Processing Software | Endoscopy | Gastroenterology | Digestive Tract | InnoVision (Shenzhen) | Shenzhen | 2024/3/26 | Ⅱ | Classical machine learning | Standard |
| NMPA | 20243210594 | Head and Neck CT Angiography Image Auxiliary Assessment Software | CT | Radiology | Head | Infervision | Beijing | 2024/3/26 | Ⅲ | Deep learning | Standard |
| NMPA | 20243210616 | Coronary Artery CT Angiography Image Vessel Stenosis Auxiliary Assessment Software | CT | Radiology | Cardiovascular | Yizhun (Zhejiang) | Lishui | 2024/3/28 | Ⅲ | Deep learning | Standard |
| NMPA | 20243210604 | Intracranial Aneurysm CT Angiography Image Auxiliary Triage Software | CT | Radiology | Head | Infervision | Beijing | 2024/3/28 | Ⅲ | Deep learning | Standard |
| NMPA | 20243210615 | Intracranial Hemorrhage CT Image Auxiliary Triage Software | CT | Radiology | Head | Deepwise (Hangzhou) | Hangzhou | 2024/3/28 | Ⅲ | Deep learning | Standard |
| NMPA | 20243210666 | Intracranial Hemorrhage CT Image Auxiliary Triage Software | CT | Radiology | Head | Dr Brain | Shenzhen | 2024/4/11 | Ⅲ | Deep learning | Standard |
| NMPA | 20243210715 | Dynamic Electrocardiogram (ECG) Analysis Software | ECG | Cardiology | Heart | Edan Instruments | Shenzhen | 2024/4/17 | Ⅲ | Deep learning | Standard |
| NMPA | 20243210771 | Coronary Artery CT Fractional Flow Reserve (FFR) Calculation Software | CT | Cardiology | Cardiovascular | United Imaging (Shanghai) | Shanghai | 2024/4/26 | Ⅲ | Deep learning | Standard |
| Guangdong | 20242210645 | Chest CT Image Processing Software | CT | Radiology | Thorax | InnerMedical | Shenzhen | 2024/5/13 | Ⅱ | Deep learning | Standard |
| NMPA | 20243211096 | Coronary Artery CT Angiography Image Vessel Stenosis Auxiliary Assessment Software | CT | Radiology | Cardiovascular | RuiXin | Shenzhen | 2024/6/11 | Ⅲ | Deep learning | Standard |
| NMPA | 20243211116 | Chest CT Image Processing and Analysis Software | CT | Radiology | Thorax | Shukun (Shanghai) | Shanghai | 2024/6/18 | Ⅲ | Deep learning | Standard |
| NMPA | 20243211109 | Fundus Image Auxiliary Diagnosis Software for Fundus Lesions | Fundus photography | Ophthalmology | Eyes | Visionary Intelligence | Beijing | 2024/6/18 | Ⅲ | Deep learning | Innovation |
| NMPA | 20243211196 | Coronary Artery CT Angiography Image Auxiliary Assessment Software | CT | Radiology | Cardiovascular | Infervision | Beijing | 2024/7/2 | Ⅲ | Deep learning | Standard |
| NMPA | 20243211300 | Focal Liver Lesion MR Image Auxiliary Assessment Software | MRI | Radiology | Liver | Yukun | Beijing | 2024/7/23 | Ⅲ | Deep learning | Standard |
| NMPA | 20243211423 | Focal Liver Lesion CT Image Auxiliary Detection Software | CT | Radiology | Liver | Sensetime | Shanghai | 2024/8/5 | Ⅲ | Deep learning | Standard |
| Beijing | 20242210408 | Medical Image Processing Software | X-ray, CT, MRI | Radiology | Multiple | Longwood Valley Medical | Beijing | 2024/8/5 | Ⅱ | Deep learning | Standard |
| Beijing | 20242210407 | Medical Image Processing Software | CT | Radiology | Multiple | Longwood Valley Medical | Beijing | 2024/8/5 | Ⅱ | Deep learning | Standard |
| NMPA | 20243211446 | Aortic Dissection CT Angiography Image Auxiliary Assessment Software | CT | Radiology | Cardiovascular | Infervision | Beijing | 2024/8/9 | Ⅲ | Deep learning | Standard |
| NMPA | 20243211562 | Electrocardiogram (ECG) Analysis Software | ECG | Cardiology | Heart | Edan Instruments | Shenzhen | 2024/8/23 | Ⅲ | Deep learning | Standard |
| NMPA | 20243211567 | Intracranial Aneurysm MR Angiography Image Auxiliary Detection Software | MRI | Radiology | Head | Fuying | Shanghai | 2024/8/23 | Ⅲ | Deep learning | Standard |
| Shanghai | 20242210288 | Bone Density CT Image Auxiliary Detection Software | CT | Radiology | Bone | Longwood Valley Medical | Beijing | 2024/8/27 | Ⅱ | Deep learning | Standard |
| Beijing | 20242210492 | Liver CT Image Processing Software | CT | Radiology | Liver | HYGEA MEDICAL | Beijing | 2024/9/12 | Ⅱ | Deep learning | Standard |
| NMPA | 20243211863 | Intracranial Hemorrhage CT Image Auxiliary Triage Software | CT | Radiology | Head | Infervision | Beijing | 2024/9/19 | Ⅲ | Deep learning | Standard |
| NMPA | 20243211866 | Pulmonary Embolism CT Angiography Image Auxiliary Triage Software | CT | Radiology | Lung | United Imaging (Shanghai) | Shanghai | 2024/9/19 | Ⅲ | Deep learning | Standard |
| NMPA | 20243211923 | Intracranial Aneurysm CT Angiography Image Auxiliary Detection Software | CT | Radiology | Head | United Imaging (Shanghai) | Shanghai | 2024/9/25 | Ⅲ | Deep learning | Innovation |
| NMPA | 20243211932 | Pulmonary Nodule CT Image Auxiliary Detection Software | CT | Radiology | Lung | Deepwise (Beijing) | Beijing | 2024/9/29 | Ⅲ | Deep learning | Standard |
| NMPA | 20243212113 | Breast X-ray Image Auxiliary Detection Software | X-ray | Radiology | Breast | United Imaging (Shanghai) | Shanghai | 2024/10/24 | Ⅲ | Deep learning | Standard |
| Jiangsu | 20242212085 | Chromosome Karyotype Analysis Software | Microscopic images | Cytogenetics | Multiple | Smiltec | Suzhou | 2024/10/29 | Ⅱ | Deep learning | Standard |
| NMPA | 20243212363 | Aortic Dissection CT Angiography Image Auxiliary Triage Software | CT | Radiology | Cardiovascular | United Imaging (Shanghai) | Shanghai | 2024/11/21 | Ⅲ | Deep learning | Standard |
| NMPA | 20243212339 | Cerebral Ischemia Image Auxiliary Assessment Software | CT, MRI | Radiology | Head | Neusoft | Shenyang | 2024/11/21 | Ⅲ | Deep learning | Standard |
| Beijing | 20242210736 | Quantitative CT (QCT) Bone Density Auxiliary Detection Software | CT | Radiology | Bone | United Imaging (Beijing) | Beijing | 2024/11/29 | Ⅱ | Deep learning | Standard |
| NMPA | 20243212456 | Intracranial Hemorrhage CT Image Auxiliary Triage Software | CT | Radiology | Head | BioMind | Beijing | 2024/12/5 | Ⅲ | Deep learning | Standard |
| Shanghai | 20242210426 | Medical Imaging Enhancement Software | MRI | Radiology | Multiple | United Imaging (Shanghai) | Shanghai | 2024/12/10 | Ⅱ | Deep learning | Standard |
| NMPA | 20243212500 | Focal Liver Lesion CT Image Auxiliary Detection Software | CT | Radiology | Liver | Pujian | Hangzhou | 2024/12/13 | Ⅲ | Deep learning | Standard |
| NMPA | 20243212624 | Coronary Artery Calcium Score Auxiliary Assessment Software | CT | Radiology | Cardiovascular | Yukun | Beijing | 2024/12/17 | Ⅲ | Deep learning | Standard |
| NMPA | 20243212606 | Electronic Lower Gastrointestinal Endoscopy Image Auxiliary Detection Software for Intestinal Polyps | Endoscopy | Gastroenterology | Colon | Huiwei | Changsha | 2024/12/17 | Ⅲ | Deep learning | Innovation |
| NMPA | 20243212627 | Pulmonary Nodule CT Image Auxiliary Detection Software | CT | Radiology | Lung | Sensetime | Shanghai | 2024/12/23 | Ⅲ | Deep learning | Standard |
| Fujian | 20252210022 | Digestive Endoscopy Medical Image Processing Software | Endoscopy | Gastroenterology | Digestive Tract | InnoVision (Xiamen) | Xiamen | 2025/1/9 | Ⅱ | Classical machine learning | Standard |
| NMPA | 20253210066 | Radiation Therapy Contouring Software | CT | Radiology | Multiple | PVmed | Guangzhou | 2025/1/10 | Ⅲ | Deep learning | Standard |
| NMPA | 20253210214 | Coronary Artery CT Angiography Image Vessel Stenosis Auxiliary Assessment Software | CT | Radiology | Cardiovascular | Yukun | Beijing | 2025/1/20 | Ⅲ | Deep learning | Standard |
| NMPA | 20253210380 | Cervical Cell Digital Pathology Image Auxiliary Diagnosis Software | Microscopic images | Pathology | Genitalia | Cells Vision | Guangzhou | 2025/2/11 | Ⅲ | Deep learning | Standard |
| NMPA | 20253210395 | Dental Panoramic Tomography Image Auxiliary Detection Software for Dental Caries | X-ray | Dentistry | Oral Cavity | DeepCare | Beijing | 2025/2/18 | Ⅲ | Deep learning | Standard |
| NMPA | 20253210397 | Head and Neck CT Angiography Image Auxiliary Detection Software | CT | Radiology | Head | Yukun | Beijing | 2025/2/18 | Ⅲ | Deep learning | Standard |
| NMPA | 20253210435 | Pulmonary Embolism CT Angiography Image Auxiliary Triage Software | CT | Radiology | Lung | Infervision | Beijing | 2025/2/26 | Ⅲ | Deep learning | Standard |
| NMPA | 20253210506 | Breast Ultrasound Image Auxiliary Detection Software | Ultrasound | Radiology | Breast | Yizhun (Beijing) | Beijing | 2025/3/6 | Ⅲ | Deep learning | Standard |
| NMPA | 20253210557 | Thyroid Nodule Ultrasound Image Auxiliary Diagnosis Software | Ultrasound | Radiology | Neck | MED Imagine | Wuxi | 2025/3/12 | Ⅲ | Deep learning | Standard |
| Shanghai | 20252210098 | Chromosome Analysis Software | Microscopic images | Cytogenetics | Multiple | Lishi Medical | Shanghai | 2025/3/14 | Ⅱ | Classical machine learning | Standard |
| NMPA | 20253210620 | Pediatric Hand X-ray Image Auxiliary Bone Age Assessment Software | X-ray | Pediatrics | Hand | Anzhikang | Hangzhou | 2025/3/21 | Ⅲ | Deep learning | Standard |
| NMPA | 20253210663 | Lung Surgery Planning Software | CT | Radiology | Lung | Infervision | Beijing | 2025/3/26 | Ⅲ | Deep learning | Standard |
| Sichuan | 20252070032 | Integrated Sleep Monitoring Workstation | Polysomnography | Sleep Medicine | Multiple | iCareTech | Chengdu | 2025/4/1 | Ⅱ | Undisclosed | Standard |
| NMPA | 20253210775 | Radiation Therapy Clinical Management Software | CT | Radiology | Multiple | Varian | Beijing | 2025/4/17 | Ⅲ | Deep learning | Standard |
| NMPA | 20253210884 | Neurosurgery Planning Software | X-ray, CT, MRI | Neurosurgery | Multiple | United Imaging (Wuhan) | Wuhan | 2025/4/29 | Ⅲ | Deep learning | Standard |
| Zhejiang | 20252211318 | Carotid Artery Image Processing Software | MRI | Radiology | Neck | Xueshi | Lishui | 2025/4/30 | Ⅱ | Undisclosed | Standard |
| Shanghai | 20252210205 | Orthodontic and Orthognathic Surgery Planning Software | X-ray, CT | Radiology | Neck | Softer Clear | Shanghai | 2025/5/22 | Ⅱ | Deep learning | Standard |
| NMPA | 20253211157 | Coronary Artery CT Angiography Image Vessel Stenosis Auxiliary Assessment Software | CT | Radiology | Cardiovascular | Keya Medical | Beijing | 2025/6/20 | Ⅲ | Deep learning | Standard |
| Beijing | 20252210686 | MR Image Processing Software | MRI | Radiology | Multiple | Wandong Medical | Beijing | 2025/6/25 | Ⅱ | Deep learning | Standard |
| Shanghai | 20252210271 | Upper Gastrointestinal Endoscopy Real-time Auxiliary Quality Control Software | Endoscopy | Gastroenterology | Digestive Tract | Fujifilm | Shanghai | 2025/6/25 | Ⅱ | Deep learning | Standard |

Note: NMPA: National Medical Products Administration;

* Included due to AI-enabling change approved in 2023.
